# The use of high-frequency ultrasound in diagnosing verrucous hemangioma

**DOI:** 10.1016/j.jdcr.2025.09.006

**Published:** 2025-09-19

**Authors:** Leonne Di Carlo Del Vecchio, Ana Luísa Pacheco Millen de Mattos, Flávia Regina Ferreira, Veridiana de Paula Santos Miranda, Vivian Castilho

**Affiliations:** Department of Dermatology, University Hospital of Taubaté, Taubaté, Brazil

**Keywords:** hemangioma, high-frequency ultrasound, keratotic lesions, pediatric dermatology, vascular lesions, verrucous

## Introduction

Verrucous hemangioma (VH), also referred to in the literature as verrucous venulocapillary malformation, is a rare congenital vascular anomaly that typically appears at birth or during childhood. It is usually unilateral and predominantly located on the lower extremities, although it may occur in other sites. Initially, the lesions appear as bluish macules, papules, and hyperkeratotic plaques that become verrucous and expand over time, without spontaneous regression.^1^, ^2^, ^3^ Histologically, these lesions show increased vascularization from the dermis to the subcutaneous tissue and have a high recurrence rate after conventional treatments.^4^^,^^5^ The most common differential diagnosis is circumscribed angiokeratoma (CA), which generally responds well to physical therapies.^6^ High-frequency ultrasound with Doppler has been increasingly used in dermatology for the diagnosis and monitoring of various diseases. Moreover, it has proven effective in differentiating between VH and CA, which motivated this report.

## Case report

**Fig 1:**
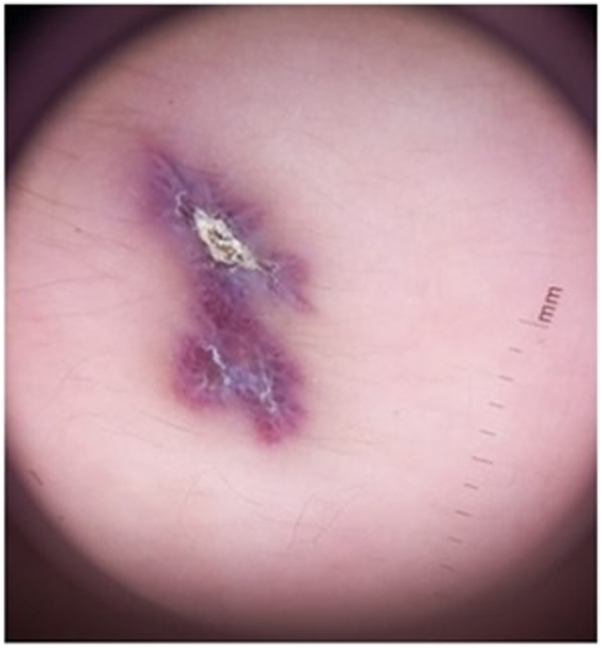
Left forearm: violet plaque, slightly elevated, with verrucous areas interspersed with areas of healthy skin.

**Fig 2:**
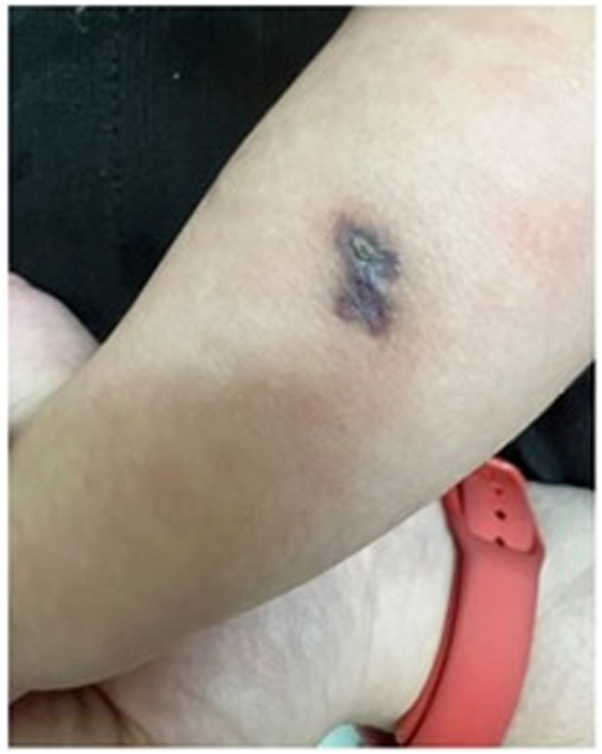
Dermatoscopy with greater detailing of the lesion.

**Fig 3:**
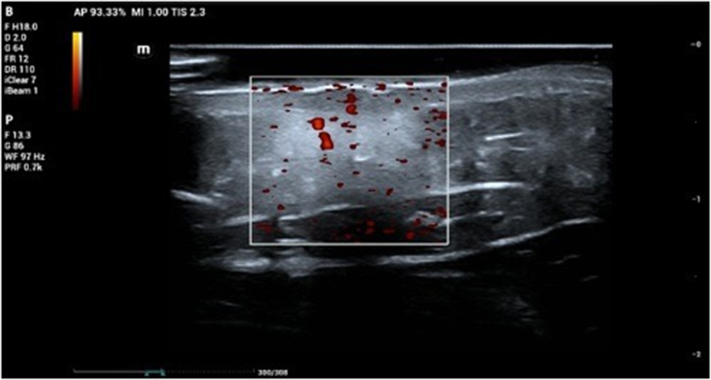
High-frequency ultrasound: dermal and subcutaneous lesion with vascular flow on power Doppler.

**Fig 4:**
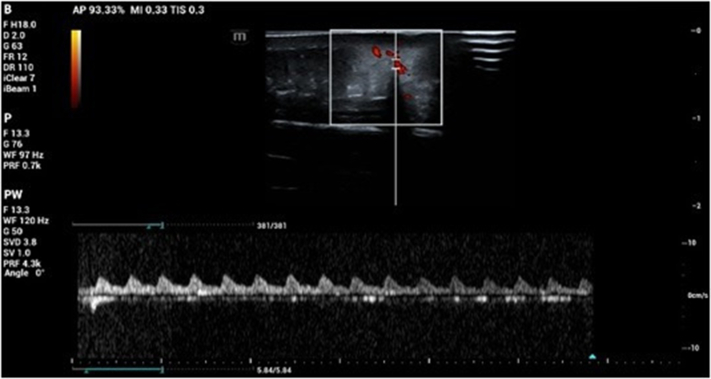
High-frequency ultrasound: Doppler spectral showing low-velocity arterial flow.

A 4-year-old female patient presented with an erythematous macule on the left forearm since birth, as reported by the mother. On physical examination, a bluish-purple plaque, slightly elevated, with areas of verrucous appearance alternating with normal skin was noted (Figs 1 and 2). There was little to no regression over the years. Clinical hypotheses included circumscribed angiokeratoma and verrucous hemangioma, and the patient was referred for high-frequency ultrasound with Doppler (23 MHz, MX7 Mindray ultrasound model). Upon examination (Color Doppler, Power Doppler, and Spectral Doppler), a hyperechoic formation with partially defined borders was observed, located in the hypodermis, with dermal involvement characterized by thickening and hypoechogenicity. The lesion was in contact with the regional muscle fascia, with no signs of muscle or fascia involvement. Doppler analysis showed predominantly arterial vascular flow in the dermis and hypodermis, with low velocity (peak systolic velocity of 5 cm/s) and average vascular density (Figs 3 and 4). Based on these findings, the hypothesis of a hemangiomatous lesion with hypodermic involvement, compatible with VH, was confirmed. The mother was informed about the benign nature of the lesion, and a watchful waiting strategy was implemented, involving regular clinical follow-up without immediate intervention. Surgical excision may be considered in the event of lesion enlargement, functional impairment, recurrent infections, or bleeding, especially when the lesion affects esthetic or psychosocial well-being.

## Discussion

Verrucous hemangioma (VH), also known as unilateral neviform hemangioma or angioqueratotic nevus, is a rare dermatological condition characterized by erythematous-blue macules that evolve into erythematous-violaceous papules and plaques with a verrucous and hyperkeratotic surface. These lesions can be aggravated by trauma or infections. The treatment of VH is challenging, with limited response to physical therapies such as cryotherapy and laser therapy, due to the extent of the lesion into the hypodermis. In many cases, deep surgical excision is required, and in some cases, skin grafts are needed.^3^, ^4^, ^5^ The main differential diagnosis is circumscribed angiokeratoma (CA), which presents as erythematous spots that evolve into papulo-nodular lesions, predominantly on the lower extremities. CA, typically congenital, does not regress spontaneously and usually responds well to treatments like electrocautery and laser. Histopathologically, VH shows angiomatous proliferation with abnormal vessels in the dermis and hypodermis, as well as acanthosis and hyperkeratosis in the epidermis. In contrast, CA involves vascular changes confined to the papillary dermis, without significant vascular proliferation, which explains the better therapeutic outcomes.^5^, ^6^, ^7^, ^8^ High-frequency ultrasound with Doppler is a valuable tool to confirm the vascular origin of the lesions and differentiate between VH and CA, as demonstrated in this case. This non-invasive method allows visualization of the depth and extent of the vessels, minimizing the need for invasive procedures like biopsies, especially in the pediatric population. Confirming the diagnosis is crucial for the proper management of keratotic vascular lesions, although there are no specific immunohistochemical markers for VH, making the clinical-pathological and ultrasonographic correlation even more essential.

## Conflicts of interest

None disclosed.
